# Breaking barriers: access to services among people living with HIV/AIDS (PLHIV) in Jharkhand – a convergent mixed methods study

**DOI:** 10.1186/s12889-026-27074-0

**Published:** 2026-03-23

**Authors:** Swati Shikha, Abhishek Kumar, Jarina Begum, Syed Irfan Ali, Badal Chandra Bhakat, Sami Akhter, Prithvish Sarkar

**Affiliations:** 1 Department of Community Medicine, Manipal Tata Medical College, Manipal Academy of Higher Education, Manipal, India; 2 Jharkhand State AIDS Control Society (JSACS), Jharkhand, India; 3 Department of Medicine, Manipal Tata Medical College, Manipal Academy of Higher Education, Manipal, India

**Keywords:** People living with HIV/AIDS, Barriers to service access, ART, Mixed methods, Jharkhand

## Abstract

**Background:**

HIV/AIDS continues to pose a significant public health challenge globally and in India, despite the availability of free antiretroviral therapy (ART) through government healthcare services. People living with HIV (PLHIV) often face multiple barriers that limit their access to essential treatment and care. Understanding these barriers—particularly those rooted in the community, health system, socioeconomic status, and personal factors—is crucial for improving service delivery and treatment adherence.

**Methods:**

This mixed-methods study was conducted over a ten-month period (February to December 2023) at an ART center in Jharkhand, India. A total of 139 PLHIV were enrolled for the quantitative component, where data were collected via a pretested structured questionnaire. Statistical analysis was performed via Jamovi (version 2.2.5) to identify associations between demographic factors and reported barriers. For the qualitative component, in-depth interviews (IDIs) were conducted with PLHIV and key ART center stakeholders. Data were analyzed via content analysis in ATLAS.ti (free trial version) and visualized via word clouds to highlight dominant themes.

**Results:**

Approximately half of the participants were male, urban residents with primary-level education. Community-level barriers prominently included stigma and fear of social exclusion. Health system barriers include drug stock-outs, lack of trust in healthcare providers, and long waiting times at ART centers. Socioeconomic challenges such as wage loss and unaffordable transportation further hinder access. Statistically significant associations were found between gender and place of residence with both health system and socioeconomic barriers. Qualitative insights complemented these findings, providing context-specific accounts of the challenges faced by PLHIV.

**Conclusion:**

PLHIV in Jharkhand face a complex interplay of community, health system, socioeconomic, and personal barriers that limit their access to ART services. These findings underscore the need for a multipronged approach that addresses structural, economic, and social determinants to improve service uptake and adherence among PLHIV.

**Trial registration:**

Not applicable, as this was an observational study without intervention.

**Supplementary Information:**

The online version contains supplementary material available at 10.1186/s12889-026-27074-0.

## Introduction

HIV/AIDS remains a major global public health concern due to its wide-ranging medical, social, and economic consequences. Since its initial identification in the late 1970s in cities such as New York, Los Angeles, and San Francisco—primarily among homosexual men—the epidemic has expanded to affect various demographic groups, including injecting drug users, sex workers, and perinatally infected children [1]. In India, the first HIV cases were reported in 1986 among female sex workers in Chennai, followed by their spread across multiple states [2]. The prevalence of HIV among adults in India is 0.20 (0.17–0.25) approximately 2.5 million people living with HIV as per the factsheets- India HIV estimates 2023 [3]. 

The HIV epidemic in India predominantly affects key populations (KPs), such as men who have sex with men, sex workers, and people who inject drugs and who are disproportionately exposed due to high-risk behaviors [4]. Curbing the epidemic requires not only effective testing and treatment services but also coordinated, compassionate, and stigma-free care for people living with HIV (PLHIV) [5]. Estimates suggest that controlling the HIV epidemic is possible if 90% of PLHIV know their status, 90% of them receive ART, and 90% on ART achieve viral suppression [6]. Aligning with this, UNAIDS “95-95-95” targets aim for 95% of PLHIV to know their status, 95% of those diagnosed must be on antiretroviral therapy (ART), and 95% of those on ART must achieve viral suppression by 2030 [7]. According to data provided by the AIDS Control Society in October 2021, 26,972 individuals in Jharkhand are HIV positive. There were 10,788 females and 16,184 males among them. With 3,126 HIV-positive people, Hazaribagh district has the highest prevalence, followed by Jamshedpur (1,822) and Ranchi (1,522). Jharkhand’s overall disease burden has steadily risen [8]. The number of PLHIV in Jharkhand is about 23,794 with prevalence of adults with HIV being 0.07 (0.06–0.09), which is below the national average and much below than high prevalent states such as Maharashtra, Karnataka, Tamil Nadu, Uttar Pradesh. Jharkhand being a tribal predominant state is different epidemiologically from many other states in India but contributes a small share to the total number of PLHIV in India. Jharkhand can therefore be called as tribal predominant low HIV burden state [3].

Despite free provision of ART under the National AIDS Control Programme in India, many PLHIV face barriers to accessing care. These include stigma, confidentiality concerns, socioeconomic constraints, and systemic inadequacies. Poor adherence to ART not only leads to treatment failure and drug resistance but also perpetuates community-level transmission [9–12]. 

Barriers span multiple levels of the socioecological model (SEM), including individual, interpersonal, institutional, community, and policy domains, and involve factors such as denial, reluctance to test, refusal of ART, discrimination, poverty, and limited access to diagnostic services [13–17]. Village Health Workers (VHWs), as community-based healthcare providers, can help foster access to HIV services by serving as a vital link between patients and healthcare facilities, thereby bridging gaps in care for people living with HIV. [18–19] Enhancing access to HIV prevention, care, and treatment services is highly important. This can be achieved by understanding the barriers to service access faced by PLHIV, the data for which is sparse from Jharkhand, hence, this study was undertaken. The incorporation of mixed method design in this study was critical to find out unexplored barriers through the qualitative component.

This study aims to identify the community, health system, socioeconomic, and personal factors that hinder access to HIV-related services among PLHIV attending an ART center in Jharkhand.

## Methods

### Study design

A convergent mixed-methods design was adopted. The quantitative component was cross-sectional, whereas the qualitative component involved in-depth interviews (IDIs) to gain deeper insights into barriers.

### Study setting and duration

The study was conducted at an ART center in Jharkhand over a 10-month period from February to December 2023.

### Participants and sampling

For the quantitative component, 139 PLHIV were selected via simple random sampling. The sample size was based on a 10% prevalence of loss to follow-up (LFU) among PLHIV. For the qualitative component, purposive sampling was used to select 30 PLHIV among those who were already the part of quantitative component of the study along with five ART center staff (medical officer, counsellor, data manager, nurse, pharmacist) for IDIs.

### Inclusion and exclusion criteria

Adults who had been diagnosed with HIV for at least six months and who provided informed consent were included. Individuals under the influence of alcohol or other substances during data collection were excluded.

### Data collection

The quantitative data were collected via a pretested structured questionnaire which consisted of closed ended questions related to already established barrier found from published literature for studies in India and was further validated by experts from National AIDS control organization and public health experts. The content validity of the barriers was established by calculating the item level content validity index (I-CVI) of each item, which is equal to the number of experts judging an item as relevant divided by the total number of subject matter experts. The quantitative data collection was integrated with qualitative data collection which was done by IDI with patients and stakeholders using a semi structured interview guide for identification of new or hidden barriers. The questionnaire as well as interview guide was developed in English language and validated before using it in the study by calculating the content validity ratio (CVR) and Scale level CVI (S-CVI). The questionnaire is attached as a supplementary file.

### Variables

The key variables included demographic characteristics and community, socioeconomic, health system, and personal-level barriers.

### Data analysis

The quantitative data were analyzed using Jamovi (version 2.2.5). Descriptive statistics in the form of frequencies and percentages and inferential statistics, including chi-square tests and Fisher’s exact tests, were used. Significance was set at *p* < 0.05. Effect sizes were calculated via Phi and Cramér’s V. Barriers were assessed using a pretested structured questionnaire consisting of multiple dichotomous (Yes/No) items under each domains. Each item in the questionnaire captured data regarding presence or absence of specific barrier. Each variable was converted into a binary composite variable where, Yes implied that participant has reported atleast one barrier in that particular domain and No meant that participant did not report any barrier in that particular domain. No summative or threshold-based scoring system was applied during analysis. Participants who reported more than one barrier within a single domain were: Counted only once under that domain as “Barrier Present”. These domains were analyzed independently and were not mutually exclusive. For each sociodemographic variable such as age group, gender, social class, residence, Chi-square tests were applied to examine associations with each binary barrier domain. The Cramér V values represent the strength of association, where values between 0.10 and 0.30 denotes weak strength of association, 0.30–0.50 as moderate and values more than 0.50 as strong association.

Qualitative data were coded and thematically analyzed using ATLAS.ti (free trial version). Themes were summarized in tabular form and visualized via word clouds. The strategy to present the finding of mixed method study was separate side by side presentation of findings.

## Results

### Participant characteristics

A total of 139 people living with HIV (PLHIV) participated in the quantitative component of the study. As shown in Table 1, the majority were male (56.5%) and resided in urban areas (60.7%). More than half (59.5%) had education above the primary level. A substantial proportion (46.4%) reported occupations categorized as “others,” including informal labor or self-employment. Most participants were married (63.7%).

**Table 1:** Sociodemographic profile of the study participants (*n* = 139)

S. No	Variable	Frequency	Percentage
1	Gender		
	Female	51	36.3%
	Male	78	56.5%
	Transgender	10	7.1%
2	Residence		
	Rural	55	39.3%
	Urban	84	60.7%
4	Education		
	Illiterate	30	21.4%
	Upto primary	26	19%
	Above primary	83	59.5%
5	Occupation		
	Unemployed	17	12.5%
	Homemaker	32	23.2%
	Daily wage worker	20	14.3%
	Service class	05	3.5%
	Others	65	46.4%
6	Marital Status		
	Married	89	63.7%
	Unmarried	27	19.6%
	Others	23	16.7%

### Barriers to Service Access

#### a) Community-level barriers

**Fig. 1:**
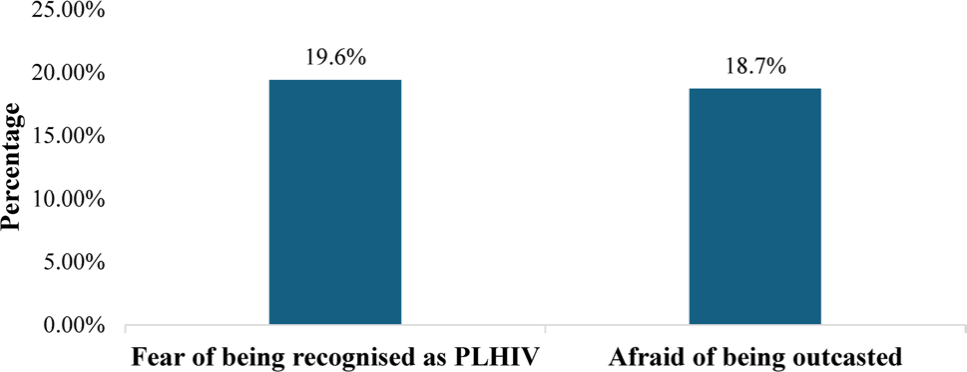
Perceived community-related barriers among people living with HIV (PLHIV). (*n* = 139)

The bar graph presented as Fig. 1 presents the various community-related barriers reported by study participants. The figure shows that 19.6% of participants reported fear of being recognized as PLHIV was a barrier, while 18.7% cited their barrier as fear of being outcasted. These findings highlight stigma and fear of social rejection as significant concerns affecting service uptake among PLHIV.

#### b) Health system-level barriers

**Fig. 2:**
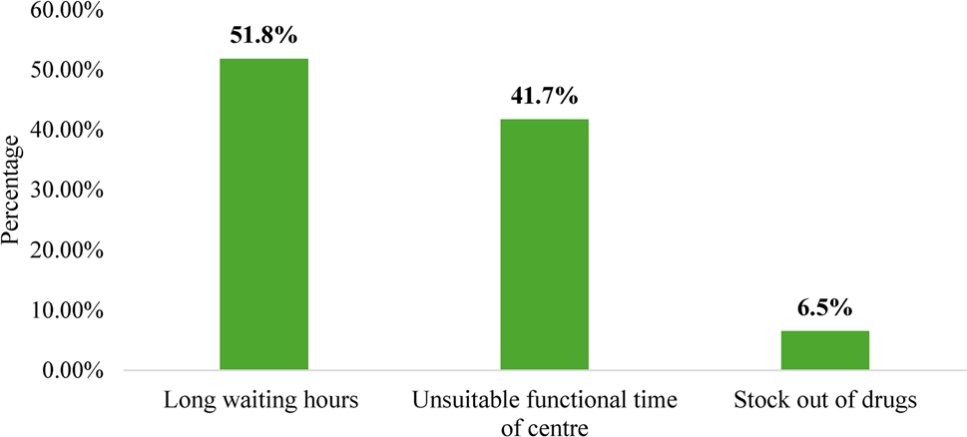
Health system-related barriers to accessing HIV services among people living with HIV (PLHIV). (*n* = 139)

Figure 2 illustrates that 51.8% of participants identified long waiting hours as a barrier to accessing HIV services, followed by 41.7% citing unsuitable functional timings of the center. A smaller proportion (6.5%) reported stock-outs of drugs as a limiting factor. These findings indicate that health system inefficiencies, particularly service delivery constraints, play a critical role in hindering access to HIV care.

#### c) Socioeconomic barriers

**Fig. 3:**
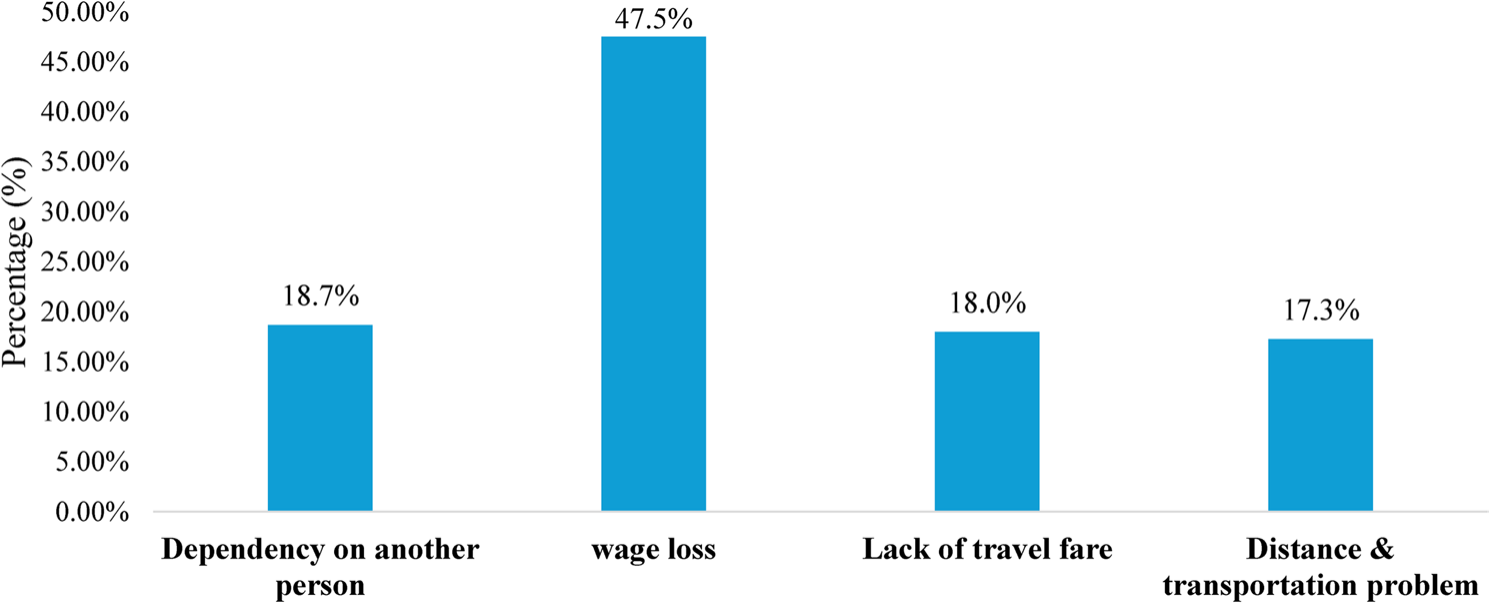
Socioeconomic barriers to accessing HIV services among people living with HIV (PLHIV). (*n* = 139)

Figure 3 depicts that wage loss emerged as the most frequently reported socioeconomic barrier (47.5%) to HIV service access. Dependency on another person (18.7%), lack of travel fare (18.0%), and transportation-related issues (17.3%) were other challenges reported. These findings suggest that financial constraints and transportation difficulties significantly impact service utilization among PLHIV.

#### d) Personal barriers

**Fig. 4:**
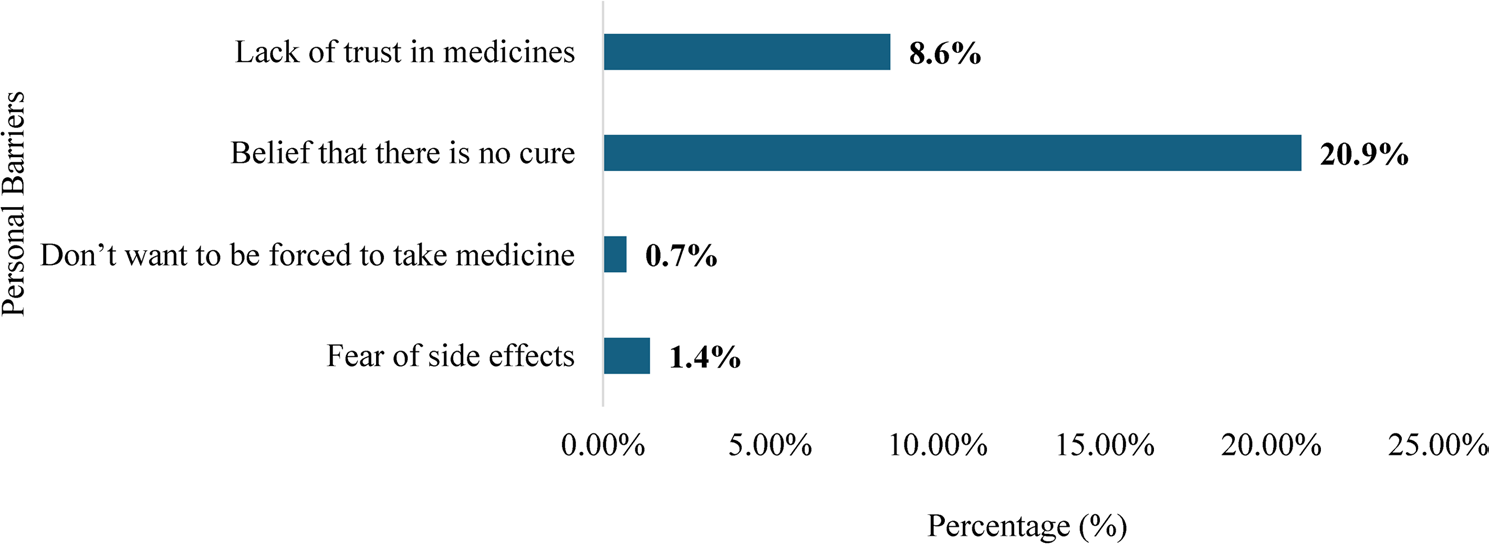
Personal barriers to accessing HIV services among people living with HIV (PLHIV). (*n* = 139)

Figure 4 depicts key personal barriers reported by participants. The most common was the belief that this disease is incurable (20.9%), followed by diminished trust in medications (8.6%). Fear of side effects (1.4%) and unwillingness to be forced to take medicines (0.7%) were few less cited hinderances. Reduced adherence to lifelong medication may be attributable to the belief that HIV is incurable which has been identified as a barrier to service access. These findings underscore how personal perceptions and misconceptions can hinder compliance with HIV care.

### Associations between Sociodemographic Factors and Barriers

Table 2 illustrates the associations between sociodemographic characteristics and perceived barriers to service access.

- Gender was significantly associated with both health system-related (*p* = 0.005, Cramér’s V = 0.276) and socioeconomic (*p* < 0.001, Cramér’s V = 0.470) barriers, with female participants reporting greater barriers.
- Residence was significantly associated with socioeconomic barriers (*p* = 0.001, Cramér’s V = 0.277), with rural participants experiencing more challenges related to cost and accessibility.
- No statistically significant associations were detected between age group or social class and community-level or health system-level barriers.

Effect size was interpreted via Cramér’s V, with values ≥ 0.3 considered to have medium-to-large clinical significance.

**Table 2:** Association between various sociodemographic variables and barriers to service access (*n* = 139)

Variables		Community Related Barrier		Association & Effect size	Health System related Barrier		Association & Effect size	Socioeconomic barriers		Association & Effect size
Age group		Yes	No		Yes	No		Yes	No	
	18–30	16 (29.1%)	39 (70.9%)	*P* = 0.169 V = 0.117	27 (49.1%)	28 (50.9%)	*P* = 0.177 V = 0.115	22 (40.0%)	33 (60.0%)	*P* = 0.066 V = 0.156
	More than 30	16 (19%)	68 (81%)		51 (60.7%)	33 (39.3%)		47 (56.0%)	37 (44.0%)	
Gender	Female	12 (23.1%)	40 (76.9%)	*P* = 0.057 V **=** 0.203	38 (73.1%)	14 (26.9%)	*P* = 0.005 V = 0.276	10 (19.2%)	42 (80.8%)	**P = < 0.001** V = 0.470
	Male	14 (18.7%)	61 (81.3%)		33 (44.0%)	42 (56.0%)		51 (68.0%)	24 (32.0%)	
	Trans gender	06 (50.0%)	06 (50.0%)		07 (58.3%)	05 (41.7%)		08 (66.7%)	04 (33.3%)	
Social Class	APL	24 (26.1%)	68 (73.9%)	*P* = 0.230 V = 0.102	55 (59.8%)	37 (40.2%)	*P* = 0.223 V = 0.103	41 (44.6%)	51 (55.4%)	*P* = 0.094 V = 0.142
	BPL	06 (12.8%)	41 (87.2%)		23 (48.9%)	24 (51.1%)		28 (59.6%)	19 (40.4%)	
Residence	Rural	10 (19.2%)	42 (80.8%)	*P* = 0.602 V = 0.044	26 (50.0%)	26 (50.0%)	*P* = 0.261 V = 0.095	17 (32.7%)	35 (67.3%)	* *P* = 0.001 V = 0.277
	Urban	20 (23.0%)	67 (77.0%)		52 (59.8%)	35 (40.2%)		09 (10.3%)	78 (89.7%)	
Residence	Rural	--	--	--	--	--	--	15 (28.8%)	37 (71.2%)	# *P* = 0.003 V = 0.256
	Urban	--	--	--	--	--	--	08 (9.2%)	79 (90.8%)	
Residence	Rural	--	--	--	--	--	--	15 (28.8%)	37 (71.2%)	**@P = < 0.001** V = 0.297
	Urban	--	--	--	--	--	--	06 (6.9%)	81 (93.1%)	

Community-related barriers: Afraid of being recognized; Health system-related barriers: Long waiting hours; Social barriers: *Dependency on another person #Lack of travel fare @Distance & transportation problem

### Qualitative findings

Insights from in-depth interviews with 30 PLHIV and 5 key ART center stakeholders provided further context for the quantitative findings. As presented in Table 3, the thematic analysis identified multiple domains of barriers, including stigma, logistical challenges, lack of supportive healthcare infrastructure, and sociocultural perceptions.

**Table 3:** Themes, domains & codes identified in in-depth interviews (IDIs) with patients (*n* = 30) and 5 key ART stakeholders

S. No	Theme	Domains	Codes
1	Services received by the PLHIV	Testing Services	Confusion about the venue Blood test, CD4 Count Urine test Saliva & sputum test Blood sugar CBC
		Diagnostic services	HIV/AIDS TB Liver diseases
		Counseling services	Preparedness counseling Available counsellors
2	Challenges faced for services	Health system related	Long queues Medicine stockouts Fear of side effects Hesitate frequent visits. Poor infrastructure Long waiting hours Timings of ART Center
		Community related	Dependent on others Social discrimination Language barrier Orphan & widow Transport from other states Hard to reach areas. Link ART Centre Homeless Job security
		Socioeconomic challenges	Wages loss Expense of travel Money crisis Long distance travel
3	Registration for ART	Knowledge of registration	Awareness Dependent on others Help of health workers Help from ICTC Information by counsellor
		TB/HIV Coinfection	Opportunistic infections TB Diabetes Hypertension Weight loss Recurrent fever Education Not aware
		2nd Line ART/ART Plus services	Other drugs 2nd Line ART Not aware Failure of ART Counseling for 2nd line ART Less adherence
4	Schemes related to PLHIV	Food Security	Antyodaya Anna Yojna (AAY) BPL Ration Card Awareness by ART Ayushman card Awareness during counseling Not aware
		Economic help	Pension by govt. PLHIV Pension by state govt. Pension by NGO Awareness of ART Help from family members. Help from friends. Help from villagers
		Any other scheme	Not aware HIV at 2017 Khusi clinic Free education
5	Discrimination in society	Social discrimination	Social outcast Nonacceptance Good society Supportive society NGO Support Acceptance in tribal groups Acceptance in MSM No water supply.
		Community outcast	Myths & beliefs Old sins Patient acceptance
		Family outcast	Lack of family support Down fall of family prestige Nonparticipation in family functions Psychiatric illness Supportive family Supportive husband Migrant Family quarrel Improvement by counseling

**Fig. 5:**
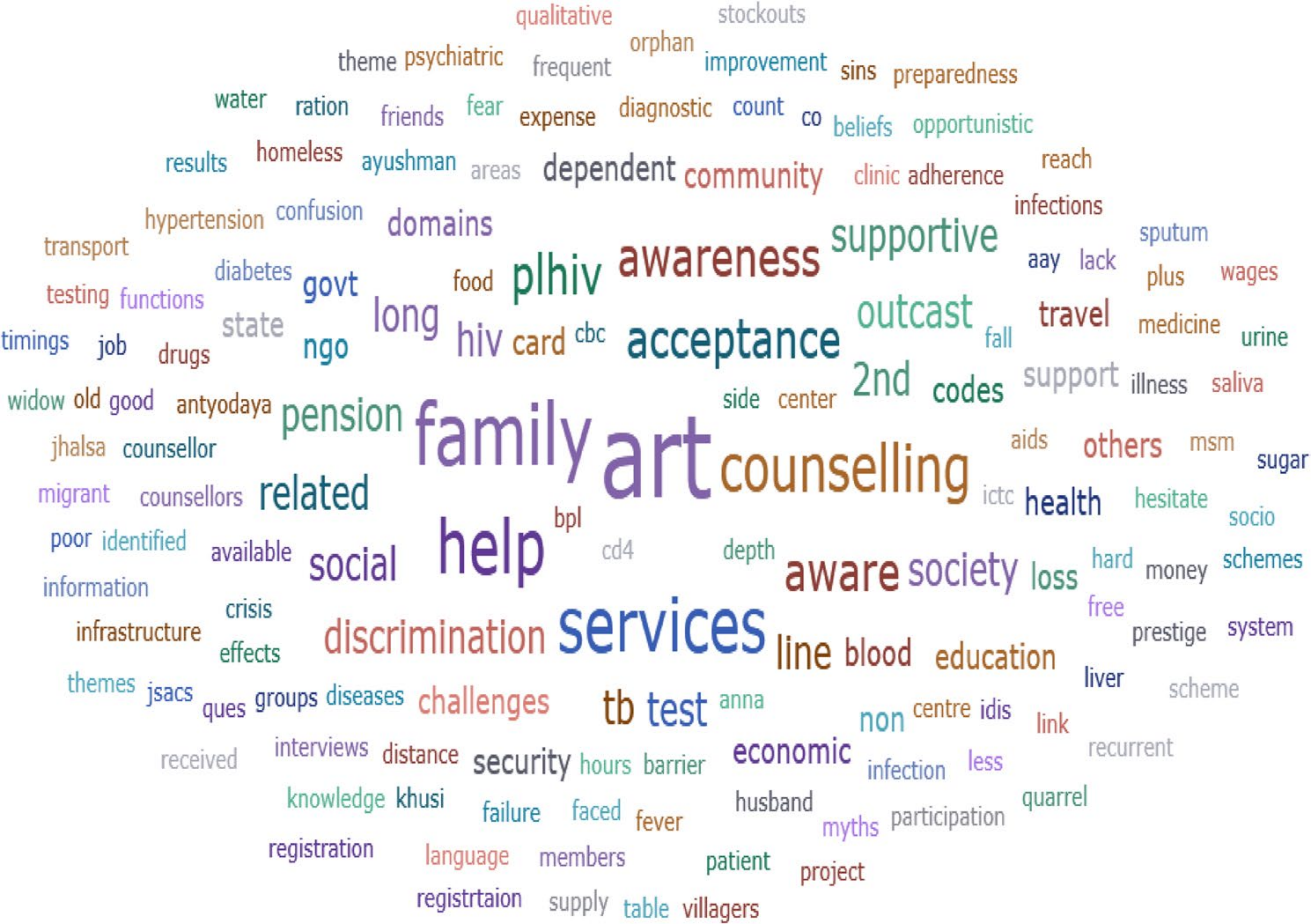
Word cloud representing key themes from in-depth interviews with people living with HIV (PLHIV). (*n* = 30) and 5 key ART stakeholders

A visual representation of the most frequently occurring codes from qualitative data identified from in-depth interviews is displayed through a word cloud (Fig. 5). Prominent themes such as “family,” “ART,” “services,” “counselling,” “awareness,” “discrimination,” and “outcast” highlight key personal, social, and systemic factors influencing access to HIV services among PLHIV and key stakeholders of ART centre. The prominence of words like “family” and “awareness” underscores the role of social support and knowledge gaps in shaping service utilization. Additionally, the appearance of terms like “discrimination” and “outcast” highlights the persistent stigma and societal barriers that contribute to service avoidance and poor treatment adherence.

## Discussion

This mixed-methods study explored the barriers to accessing HIV-related services among people living with HIV (PLHIV) attending an ART center in Jharkhand. Both the quantitative and qualitative findings identified a range of personal, health system-related, socioeconomic, and community-level barriers that hinder service utilization.

Among the most frequently reported barriers were prolonged waiting times at ART centers and financial losses due to missed work, which is consistent with findings from similar settings. Community-level factors such as stigma, fear of social recognition, and perceived discrimination continue to exert a strong influence on healthcare-seeking behaviors. This aligns with a study by Nair et al. [20] in Bihar, which revealed that stigma and disclosure concerns—especially in rural settings—significantly impeded access to services. The key themes of that study included involuntary disclosure, community-level misconceptions, and a lack of universal precaution practices among providers—factors that are also relevant to the present findings.

Charles et al. [21] reported high levels of stigma among PLHIV, including personal stigma, negative self-image, and worries about disclosure. These aspects were echoed in other studies as well [22–24], where internalized stigma which developed because of counselling or fear of being judged was a recurring theme. Interestingly, perceived stigma was often found to be greater than the actual stigma experienced [23], indicating that fear-based avoidance may be as critical as experienced discrimination. Stigma is a significant barrier to compliant HIV treatment [25]. 

Our study also highlighted personal-level barriers, with approximately 21% of participants believing that HIV is incurable and 8.6% expressing doubts about the efficacy of ART. A small proportion also feared medication side effects. These findings align with findings from a study conducted in Maharashtra [26], which underscored the impact of sociocultural factors, economic instability, and personal perceptions on ART adherence and clinical follow-up. That study also revealed that strained doctor‒patient communication and limited counseling time contributed to service gaps—factors similarly noted by participants in our qualitative interviews.

As far as the transgenders are concerned, it was observed in our study that 58.3% of them faced health system related barriers and 66% of them faced socio-economic barrier. The study by Chakrapani Venkatesan [27] demonstrates that intersecting stigmas related to transgender identity, HIV status, and sex work substantially impede HIV care engagement among transgender women living with HIV (TGWLH) in India. Enacted and anticipated stigma, coupled with lack of gender-affirming practices such as misgendering in healthcare settings, contributed to delayed ART initiation and care disengagement. Stigma within transgender women (TGW) communities further influenced engagement by promoting concealment of HIV status and co-occurring conditions, including depression and alcohol use, reflecting interacting psychosocial vulnerabilities. In contrast, supportive and affirming physicians, counsellors, and peer outreach workers within ART centres facilitated ART initiation, adherence, and retention. TGW communities also functioned as important resilience resources, offering emotional and practical support that reduced the impact of discrimination. These findings indicate the need for public health interventions that reduce intersecting stigmas, strengthen gender-affirming and culturally competent HIV care, integrate mental health services, and reinforce community-based support mechanisms. Another study by W Lodge II et al. [28] highlights persistent barriers and facilitators influencing ART adherence among TGW living with HIV in urban India. Despite free and immediate ART access under the “test and treat” policy, adherence is undermined by structural and social determinants. Guided by intersectionality and syndemic theory, findings show that poverty amplifies co-occurring stressors, including housing insecurity, food insufficiency, and mental health challenges, while intersecting stigma related to gender identity, HIV status, and socioeconomic marginalization further disrupts engagement in care. In contrast, individual and community empowerment, along with inclusive government programmes, supported sustained ART use. From a public health perspective, integrating economic support, stigma-reduction strategies, and TGW-affirmative services into HIV programmes is essential to improve ART adherence, achieve viral suppression, and advance equity within India’s HIV response.

As per the findings of a study by Heylen et al. [11] suboptimal adherence to ART contributes to drug resistance, treatment failure, and onward HIV transmission. In the study by Heylen et al., people living with HIV in Karnataka reported taking only 68% of prescribed doses, with adherence barriers widespread across individual, social, and clinic-related domains. Better adherence was associated with disclosure to adult household members, use of multiple adherence strategies, perceived benefits of ART, and longer treatment duration, while fear of stigma related to disclosure to friends and workplaces was negatively associated. These findings suggest that individual barriers such as forgetfulness may be addressed through simple strategies, whereas stigma-related barriers require family- and community-level interventions.

The cumulative evidence indicates that while services are available, multiple barriers—both perceived and actual—diminish their accessibility and effectiveness. Strengthening trust in the healthcare system, reducing waiting times, addressing socioeconomic vulnerabilities, and tackling persistent stigma are essential steps for improving service utilization.

## Conclusion

This study concludes that while the majority of PLHIV attending the ART center benefit from available services and demonstrate regular follow-up, several barriers still impede equitable access. These include:

- Community-related barriers: fear of being recognized and stigmatized.
- Health system-related barriers: long waiting queues, stock-outs of medicines, inadequate counseling time, and language barriers.
- Socioeconomic barriers: wage loss, transportation challenges, and delays in availing of social welfare schemes.
- Personal barriers: lack of trust in treatment effectiveness and misconceptions about disease curability.
- On regression analysis, more of female participants were found to be experiencing both health system-related and socioeconomic barriers and socioeconomic barriers were more common among participants from rural background.

Despite the efforts of ART center staff and programme implementers, addressing these multilevel barriers remains critical for achieving the UNAIDS 95-95-95 targets and ending HIV/AIDS as a public health threat by 2030.

### Definition of terminology used

Illiterate- A person, who can neither read nor write or can only read but cannot write in any language, is treated as illiterate [29]

### Limitations

This study was carried out among PLHIV from few districts of Jharkhand, which may limit the generalizability of the results to other regions of the country. Additionally, due to resource constraints, participants were recruited from those registered at the ART center, potentially excluding PLHIV who are not engaged with formal healthcare services. Given the sensitive nature of HIV status, there is a possibility that some participants may have underreported the barriers they faced in accessing services. Furthermore, as key populations were not sampled following the Probability Proportional to Size approach, certain high-risk groups such as MSM, transgender individuals, and others may have been underrepresented in this study.

## Supplementary Information

Supplementary Material 1.

## Data Availability

The dataset generated from the study is available with the corresponding author on reasonable request.

## Abbreviations

PLHIV: People living with Human Immunodeficiency Virus
ART: Anti Retroviral Therapy
HIV: Human Immunodeficiency Virus
AIDS: Acquired Immunodeficiency Syndrome
IDI: In-depth interview
KP: Key population
UNAIDS: Joint United Nations Programme on HIV/Aids (UNAIDS)
SEM: Socioecological model
VHW: Village health worker
LFU: Loss to follow up
MTMC: Manipal Tata Medical College
APL: Above poverty line
BPL: Below poverty line
ICTC: Integrated counselling and testing centre
TB: Tuberculosis
AAY: Antyodaya anna yojana
NGO: Nongovernmental organization
MSM: Men Sex with Men
JSACS: Jharkhand state AIDS control society
NACO: National AIDS control organization
TGWLH: Transgender women living with HIV
TGW: Transgender women
I-CVI: Item level Content Validity Index
S-CVI: Scale level Content Validity Index
CVR: Content Validity Ratio
