# Supplementary material for: Breaking barriers: access to services among people living with HIV/AIDS (PLHIV) in Jharkhand – a convergent mixed methods study
Source: BMC Public Health. 2026 Mar 23;26:1417. doi: 10.1186/s12889-026-27074-0 (PMC13130708; doi:10.1186/s12889-026-27074-0)
Supplement: Supplementary file 1 — Supplementary Material 1. [file 12889_2026_27074_MOESM1_ESM.pdf]

Questionnaire

I. Sociodemographic profile

S. No.	Variable	Response
1	Patient Number	
2	Age<br>a) Less than/equal to 30 years (18 – 30 years)<br>b) 31 – 60 years<br>c) More than 60 years	
3	Gender<br>a) Male<br>b) Female<br>c) Transgender	
4	Residence<br>a) Rural<br>b) Urban	
5	District of Residence	
6	Education<br>a) Illiterate<br>b) Upto Primary (upto class 5)<br>c) Above primary	
7	Occupation<br>a) Unemployed<br>b) Service class<br>c) Daily wage worker<br>d) Home maker<br>e) Others (Specify)	
8	Marital status<br>a) Married<br>b) Unmarried<br>c) Others (Specify)	
9	Total Annual Income of the family	
10	Social Class of the family	a) BPL<br>b) APL

II. Perceived barriers to HIV Care

S. No.	Variable	Response
1	Do you visit ART Centre regularly<br>a) YES<br>b) NO	
2	How frequently do you visit the ART centre<br>a) Once a month<br>b) Once in 2 months<br>c) Ill-defined frequency	
3	**Community related barriers** towards HIV care services, please tick the most appropriate response for each of the following:<br>a) Fear of being recognized	▪ YES<br>▪ NO
	b) Stigma and discrimination towards PLHIV	▪ YES<br>▪ NO
4	**Health system related barriers** towards HIV care services, please tick the most appropriate response for each of the following:<br>a) Lack of trust in health system	▪ YES<br>▪ NO
	b) Belief that there is No cure	▪ YES<br>▪ NO
	c) Stock out of drugs	▪ YES<br>▪ NO
	d) Unsuitable working hour of service provider centre	▪ YES<br>▪ NO
	e) Long waiting hours at the centre	▪ YES<br>▪ NO
	f) Don't want to be forced to take medicines	▪ YES<br>▪ NO
	g) Fear of side effects of drugs	▪ YES<br>▪ NO

	h) Satisfaction with the grievance redressal mechanism	▪ Fully Satisfied<br>▪ Satisfied<br>▪ Somewhat satisfied<br>▪ Not Satisfied
	i) Interaction with the counsellor	▪ Satisfactory<br>▪ Un-satisfactory
	j) Any follow-up by the health worker	▪ YES<br>▪ NO
5	**Social barriers** towards HIV care services, please tick the most appropriate response for each of the following:<br>a) Dependency on another person to accompany to ART Centre	▪ YES<br>▪ NO
	b) Wage loss on the day of visit	▪ YES<br>▪ NO
	c) If YES in the above Q 5(b), then how much	
	d) Lack of travel fare	▪ YES<br>▪ NO
	e) Distance and transportation problem	▪ YES<br>▪ NO
6	Any other perceived barrier to HIV care (If YES, Specify)	
7	Any suggestions for improvement of services at the ART centre	

<u>IDI Questions with Probes</u>

1) Mention the services being received by the PLHIV
   Probes for discussion:
   a. Diagnostic services
   b. Testing services
   c. Counselling services

2) Challenges being faced for service access
   Probes for discussion:
   a. Health system related challenges
   b. Community related challenges
   c. Socio-economic challenges

3) Knowledge about the process of getting registered for ART
   Probes for discussion:
   a. Accompanied by any health worker
   b. Knowledge about TB/HIV Co-infection
   c. Knowledge about 2nd line ART/ART Plus

4) Brief about some schemes related to PLHIV
   Probes for discussion:
   a. Food security
   b. Economic help (Pension Scheme)
   c. Any other related scheme

5) Discrimination faced after being diagnosed with HIV/AIDS
   Probes for discussion:
   a. Social discrimination
   b. Community outcast
   c. Family outcast
